# A comparative study of thoracoscopic and open surgery of congenital diaphragmatic hernia in neonates

**DOI:** 10.1186/s13019-019-0938-3

**Published:** 2019-06-26

**Authors:** Jing Qin, Yongying Ren, Deliang Ma

**Affiliations:** 1 Departments of Anesthesiology, Linyi Central Hospital, No.17 Jiankang Road, Yishui County, Linyi, 276400 Shandong Province China; 2 Departments of Medical Oncology, Linyi Central Hospital, No.17 Jiankang Road, Yishui County, Linyi, 276400 Shandong Province China

**Keywords:** Congenital diaphragmatic hernia, Neonates anesthesia, Open surgery, Thoracoscopy

## Abstract

**Background:**

An increasing number of hospitals have carried out neonatal thoracoscopic assisted repair of congenital diaphragmatic hernia (CDH).

**Methods:**

The 26 cases received thoracoscopic-assisted repair (observation group) and 44 cases open repair (control group). General anesthesia was performed with endotracheal intubation using a trachea cannula without cuff. The general preoperative data, intraoperative hemodynamic parameters, intraoperative surgical conditions, postoperative complications, postoperative recovery condition, postoperative survival rate and recurrence rate were recorded.

**Results:**

The intraoperative mean arterial pressure and heart rate at each time point in observation group were more stable and effective than those in control group (all *P* <  0.001). The number of manual ventilation, SpO_2_ < 90% and hypercapnia cases were significantly lower than those in control group (all *P* <  0.05). Intraoperative bleeding, incision length and operation duration were significantly lower in observation group compared with control group (all *P* <  0.001). No significant differences were seen between the two groups in postoperative complications including pulmonary infection, incision infection, pulmonary hypertension, hemorrhage, and scleredema (all *P >* 0.05). The duration of postoperative mechanical ventilation, antibiotic use and hospitalization in observation group was significantly shorter than those in control group (all *P <* 0.05). There was no significant difference in postoperative survival rate and recurrence rate between the two groups (both *P >* 0.05).

**Conclusion:**

The intraoperative hemodynamic parameters of CDH repair under thoracoscopy were more stable, the duration of postoperative mechanical ventilation, antibiotic use and hospitalization were shortened, and the therapeutic effect was better.

## Background

The incidence of congenital diaphragmatic hernia (CDH) is about 1:2, 500–1:3, 500. It is a disease caused by incomplete closure of the fetal diaphragm muscle in embryonic development and the entry of abdominal organs into the thoracic cavity due to diaphram hypoplasia, which results in abnormal anatomical relationships. Most CDHs occur on the left side, most of which don’t form a hernia sac and commonly contain abdominal organs [1, 2]. Due to the occupation of space by the hernia contents in the thoracic cavity during intrauterine development, the affected side will squeeze the contralateral lung, resulting in the restricted contralateral lung development and varying degrees of pulmonary dysplasia or pulmonary hypertension [3, 4]. Some children will have intestinal malrotation, affecting the development of the digestive system and resulting in abnormal intestinal development. In addition, CHD may also present along with other system malformations, such as central nervous system abnormalities, esophageal atresia, omphalocele, cardiovascular defects and other congenital malformation of various organ systems. Therefore, prenatal and postnatal diagnosis, perioperative treatment and early surgery are crucial to the recovery of children with CDH.

Reduction of hernia contents and repair of diaphragmatic hernia after the birth of CDH children is the standard treatment. The traditional surgical approach is a transabdominal diaphragmatic herniorrhaphy. Although it has a good effect, the traditional operation has the disadvantages of long duration, large trauma, long hospital stay and high rate of complication. In recent years, thoracoscopic repair of diaphragmatic hernia, a minimally invasive surgery, has become the main method for surgical treatment of CDH [5, 6]. Compared with traditional transabdominal surgery, thoracoscopic CDH repair has advantages including less damage, better visual field and convenient operation, and is increasingly used in clinical practice. Thoracoscopic diaphragmatic herniorrhaphy is an effective suture of the defect area of the posterior diaphragm wall in children under thoracoscope, which can reduce the length of incision and make the suture more accurate. It not only has a higher surgical effect, but also can significantly reduce the pain of children, and reduce the incidence of complications and recurrence rate. However, children with CDH are younger, have low body weight, have varying degrees of pulmonary dysplasia, increased pulmonary vascular resistance, and may also have heart or other organ system malformations. Therefore, diaphragmatic herniorrhaphy under thoracoscopy is a major challenge for anesthesiologists [7, 8]. In addition, unfavorable factors such as the intraoperative pressure of artificial pneumothorax on lung tissue under thoracoscopy as well as the requirement of a certain degree of lung collapse on the surgical side to maintain the surgeon’s visual field, make anesthesia management much more difficult during surgery [9]. Therefore, the perioperative management of thoracoscopic congenital hernia repair requires comprehensive intraoperative monitoring and postoperative follow-up in addition to anesthesia management during transabdominal diaphragmatic hernia repair.

There are not many reports on perioperative anesthesia management of CDH thoracoscopic surgery. Therefore, this paper reviewed the perioperative anesthesia management experiences of 70 children with CDH in our hospital from January 2015 to January 2018, and compared the influence of the two surgical methods on perioperative management and prognosis.

## Methods

### General data

This study included 82 children with CDH who were admitted to our hospital from 2015 to 2018. The children diagnosed with CDH were evaluated and treated with tracheal intubation ventilator support before cord clamping. Twelve patients died of severe disease before surgery. After adjusting electrolytes, acid-base balance, coagulation function and circulatory support, the other 70 cases underwent diaphragmatic herniorrhaphy. Among them, 26 cases received thoracoscopic-assisted diaphragmatic hernia repair (observation group) and 44 cases received open diaphragmatic hernia repair (control group), according to the opinions of the patient’s family and the patient’s condition. The family of each patient signed the informed consent form, and the study was approved by the Ethics Committee of Linyi Central Hospital (No. 2015013001).

Inclusion criteria: Comply with Canadian Clinical Practice Guide 2018: Diagnosis and Management of Congenital Diaphragmatic Hernia; Apgar score > 4; recover vital signs after rescue in spite of ischemia and hypoxia [10, 11].

Exclusion criteria: Newborns who failed to be rescued; children with congenital central nervous system malformation, serious digestive system malformation, severe cardiovascular malformation, such as congenital heart disease, including atrial septal defect, ventricular septal defect, patent ductus arteriosus, patent foramen ovale, and various degrees of pulmonary hypertension; children with non-cyanotic congenital heart disease with small defects; and children with more complicated, cyanotic congenital heart disease who were not suitable for thoracoscopic surgery.

### Intraoperative management

General anesthesia was performed with endotracheal intubation using the trachea cannula without cuff (Xintaike, China). At birth, tracheal intubation, mechanical ventilation and nasogastric tube placement were performed before umbilical cord disconnection, and then the child was transferred to pediatric surgical intensive care unit for ventilator therapy. A series of preoperative examinations were performed, including echocardiography, arterial blood gas and electrolytes, and chest radiography. After the child entered the operating room, the trachea catheter was connected to the anesthetic machine (Bori 700D, Probe, Shenzhen) for mechanical ventilation. After confirming that the tracheal tube was placed in the trachea, pressure support mode was utilized. Inspiratory pressure and respiratory frequency were adjusted according to the end-tidal carbon dioxide (ETCO_2_) value and pulse oxygen saturation (SpO_2_). And at the same time, invasive arterial manometry and deep vein catheterization via internal jugular vein were performed. Anesthesia-inducing drugs include 0.02 mg/kg atropine (Hubei Xinrunde Chemical Co., Ltd., Hubei), 0.1 mg/kg midazolam (Jiangsu Nhwa Pharmaceutical Co., Ltd., Jiangsu), 2 mg/kg propofol (Fresenius Kabi, Beijing), 0.2 mg/kg Atracurium Cisatracurium besilate (Chongqing Saipunasi Technology Co., Ltd., Chongqing), and 1–2 μg/kg fentanyl (Yichang Humanwell Pharmaceutical Co., Ltd., Fujian). Anesthesia was maintained by inhalation of sevoflurane (2–2.5%) and intravenous pumping of remifentanil (0.1–0.3 μg/kg/min). The ventilator controlled respiration using pressure control mode. The inspiratory pressure was 15–20 cmH_2_O, the respiratory rate was 20–24 times / min, and the breathing ratio was 1:2. Depending on the situation, a positive end expiratory pressure of 1–4 cmH_2_O could be used. The intraoperative parameter was adjusted according to ETCO_2_ which should be maintained at 35–60 mmHg. The intraoperative artificial pneumothorax pressure was set at approximately 2–6 mmHg, and the gas flow rate was approximately 1.5–2 L/min. When SpO_2_ < 90% or ETCO_2_ > 60 mmHg, manual manipulation was performed to maintain the respiratory rate at 20–35 times / min, the peak inspiratory pressure at < 30 mmHg, and the mean airway pressure at < 6 mmHg until the conditions above were improved. Endotracheal catheters of all patients were reserved and patients were transferred to pediatric surgical intensive care unit for continued ventilator support.

### Operation methods

Control group: The children were given general anesthesia, 5–8 cm transverse incision was made under the rib margin of the left upper abdomen. After opening the abdomen, the internal organs entering the thoracic cavity were repositioned, and the defect margin was sutured by intermittent mattress suture after fully exposing the defect area of the diaphragm. If there was a hernia sac, it should be resected. After the operation, according to the actual situation of the child, determine whether the drainage tube needs to be detained, close the chest and return to the intensive care unit.

Observation group: The children underwent general anesthesia with tracheal intubation. The children took the head high and feet low, the right side lying position, and the left upper arm raised to ensure the elevation of the subscapular angle to the fifth intercostal plane. Three Trocars were placed in the middle of the umbilical cord and in the left and right upper abdomen. Then, with the help of pneumothorax pressure and manipulation forceps, the hernia contents were repositioned to the abdominal cavity, and the diaphragm defect was fully exposed in order to observe the size of the diaphragm defect. If there is a hernia sac in the child, push it to the abdominal side, and then use 2–0 non-absorbable needle suture to suture the defect diaphragm intermittently. The patch uses a new type of lightweight ultrapro hernia system (UHS) device to suture the defect from both sides of the small tension to the middle. After suturing, the operating instruments were pulled out, the incision was sutured, and the tracheal intubation was removed after the patient was awake and in stable situation.

### Observation indicators

Main observation indicators: Postoperative respiratory complications, incidence of pulmonary hypertension, postoperative mechanical ventilation duration, antibiotic use duration, hospital stay, postoperative survival rate and recurrence rate.

Secondary observation indicators: Number of cases of cardiac malformation combined with pulmonary hypertension, preoperative mechanical ventilation adjusted blood gas, and intraoperative manual ventilation.

Successful operation: X-ray examination showed complete repair of hernia sac; the whole lung volume and effective lung volume decreased while the residual volume increased; pulmonary artery pressure was normal.

Postoperative recurrence: Complications such as hernia sac protrusion, pulmonary volume increase, cardiovascular malformation, etc.

The lung to head ratio (LHR) is an indicator for assessing and predicting fetal prognosis. LHR = (right lung long diameter × right lung short diameter) / head circumference. If LHR > 1.4, the prognosis for the child is good; a LHR < 1.0 indicates severe CDH and poor prognosis.

An arterial blood PH < 7.3 or PaCO_2_ > 60 mmHg indicates hypoxia, acidic blood and severe diaphragmatic hernia.

### Statistical method

SPSS 22.0 statistical software was used for analysis. The measurement data were expressed as mean ± standard deviation ($\overline{x}$ ± sd). Two sample t test was used for inter-group comparison, and one-way ANOVA was used for intra-group comparison at different time points. Enumeration data were expressed as case / percentage (*n*/%) and tested by χ^2^ test or Fisher’s exact test. *P <* 0.05 was considered statistically significant.

## Results

### General data

There was no significant difference in preoperative data between the two groups including gender, age, LHR < 1.0 cases, preoperative tracheal intubation cases, preoperative PH < 7.3 cases, preoperative PaCO_2_ > 60 mmHg cases, pulmonary hypertension cases and cardiac malformation cases (all *P >* 0.05, Table 1).

**Table 1:** Comparison of general information (*n*, %)

Group	Observation group (*n* = 26)	Control group (*n* = 44)	t/χ^2^	*P*
Gender (male / female)	10/16	19/25	0.150	0.689
Age (day)	15.3 ± 2.1	14.7 ± 2.8	0. 348	0.946
LHR < 1.0	6 (23.08)	11 (25.00)	0.033	0.856
Preoperative tracheal intubation	24 (92.31)	40 (90.91)	0.041	0.840
Preoperative PH < 7.3	20 (76.92)	34 (77.27)	0.001	0.973
Preoperative PaCO_2_ > 60 mmHg	15 (57.69)	27 (61.36)	0.092	0.762
Cardiac malformation	5 (19.23)	9 (20.45)	0.015	0.902

*LHR* the lung to head ratio

### Intraoperative hemodynamic changes

**Fig. 1:**
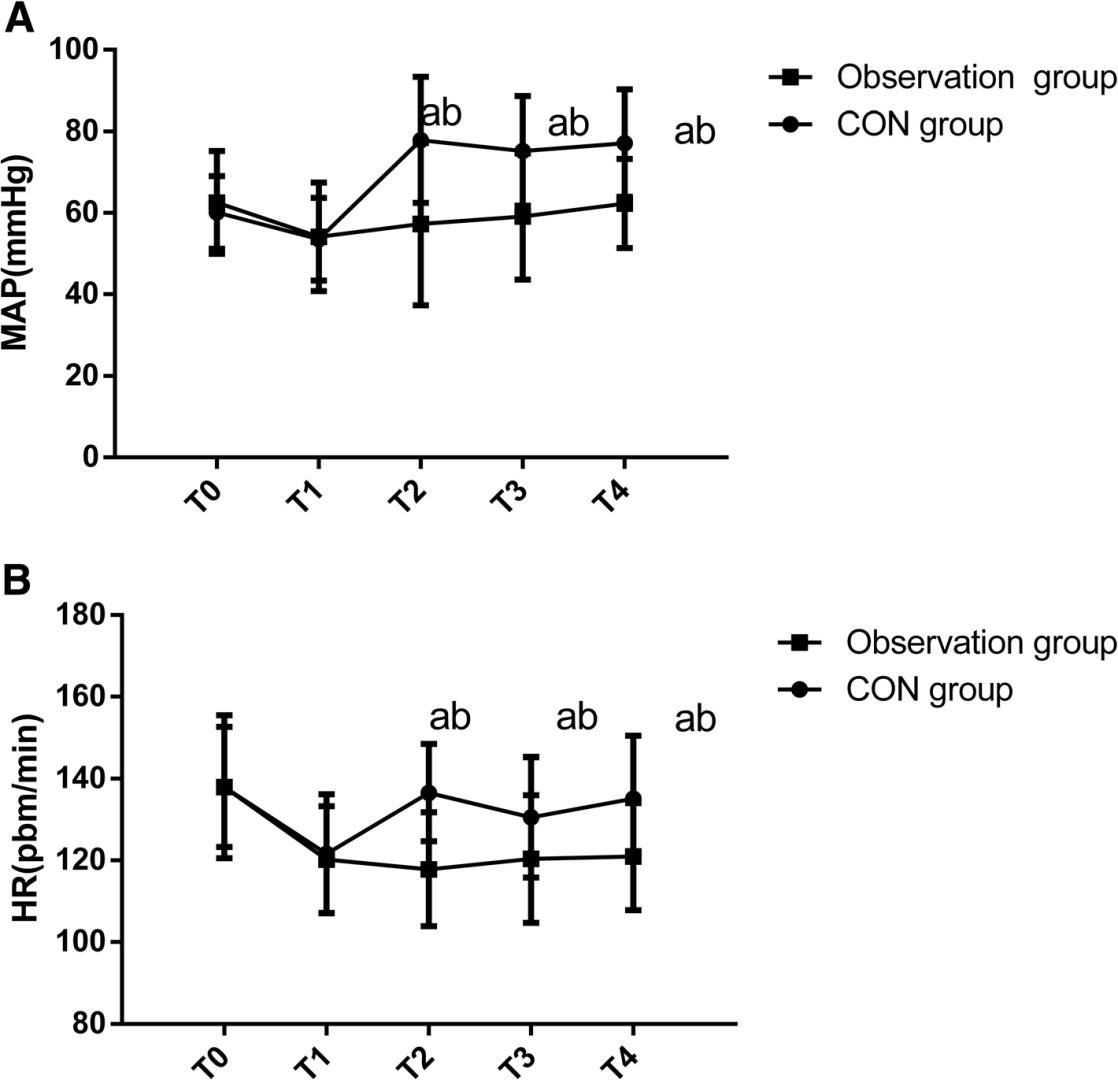
Intraoperative hemodynamic changes in HR (**a**) and MAP (**b**). ^a^*P* < 0.001, compared within the same group after induction; ^b^*P* < 0.001, compared with observation group. T0, pre-induction; T1, post-induction; T2 T3, T4 and T5 indicated 1 min, 10 min, 60 min and 120 min after surgery, respectively; CON, control; HR, heart rate; MAP, mean arterial pressure

Compared with post-induction, MAP and HR in the control group were significantly increased at 10 min, 60 min, and 120 min after surgery (all *P <* 0.001). However, in the observation group, there was no significant difference in MAP and HR between post-induction and each time point after surgery (both *P >* 0.05), indicating that anesthesia was stable and effective. MAP and HR of each time point in the control group were significantly higher than those in the observation group (both *P <* 0.001, Fig. 1, Table 2).

**Table 2:** Intraoperative hemodynamic changes

	MAP (mmHg)		HR (time / min)	
	Control group (*n* = 44)	Observation group (*n* = 26)	Control group (*n* = 44)	Observation group (*n* = 26)
Before induction	60.13 ± 8.94	62.56 ± 12.62	137.96 ± 14.65	137.98 ± 17.45
After induction	53.56 ± 10.16	54.12 ± 13.37	121.64 ± 14.57	120.22 ± 13.08
After surgery				
10 min	77.91 ± 15.47^ab^	57.26 ± 19.94	136.56 ± 11.87^ab^	117.83 ± 13.95
60 min	75.22 ± 13.44^ab^	59.12 ± 15.47	130.54 ± 14.76^ab^	120.35 ± 15.63
120 min	77.12 ± 13.14^ab^	62.33 ± 10.95	135.13 ± 15.35^ab^	120.92 ± 13.15

^a^*P* < 0.001, compared within the same group after induction; ^b^*P* < 0.001, compared with observation group. *HR* heart rate, *MAP* mean arterial pressure

### Comparison of intraoperative conditions

The number of manual ventilation cases, SpO_2_ < 90% cases and hypercapnia cases in the observation group were significantly lower than those in the control group (all *P <* 0.05). Intraoperative bleeding, incision length and operation duration were also significantly lower in the observation group compared to the control group (all *P <* 0.001, Table 3).

**Table 3:** Comparison of intraoperative conditions

Group	Observation group (*n* = 26)	Control group (*n* = 44)	t/χ^2^	*P*
Manual ventilation (case)	4 (15.38%)	17 (38.64%)	4.207	0.040
SpO_2_ < 90% (case)	3 (11.54%)	15 (34.09%)	4.351	0.037
Hypercapnia (case)	2 (7.69%)	12 (27.27%)	3.916	0.048
Operation duration (min)	137.230 ± 23.180	157.273 ± 19.380	4.970	< 0.001
Intraoperative bleeding (mL)	247.230 ± 12.390	283.223 ± 14.290	10.68	< 0.001
Incision length (cm)	3.290 ± 0.730	6.281 ± 1.282	10.89	< 0.001

*SpO*_*2*_ pulse oxygen saturation

### Comparison of the complications

No significant differences were seen between the two groups in postoperative complications including pulmonary infection, incision infection, pulmonary hypertension, hemorrhage, and scleredema (all *P >* 0.05, Table 4). There was no significant difference in postoperative survival rate or recurrence rate between the two groups (both *P >* 0.05). In the control group, 1 case died after open diaphragmatic hernia repair, in which no improvement was observed due to left side fluid pneumothorax and cardiopulmonary insufficiency.

**Table 4:** Comparison of the complications (*n*, %)

Group	Observation group (*n* = 26)	Control group (*n* = 44)	Fisher/χ^2^	*P* (Fisher)
Pulmonary infection	3	9	0.902	0.514
Incision infection	2	7	0.971	0.468
Pulmonary hypertension	3	7	0.251	0.734
Hemorrhage	0	5	3.136	0.150
Scleredema	1	5	1.162	0.401
Survival rate	26 (100.00)	43 (97.73)	0.599	0.439
Recurrence rate	3 (11.54)	5 (11.36)	0.031	> 0.999

### Postoperative recovery comparison

The duration of postoperative mechanical ventilation, antibiotic use and hospitalization in the observation group was significantly shorter than those in the control group (all *P <* 0.05, Table 5).

**Table 5:** Comparison of postoperative recovery ($\overline{x}$ ± sd)

Group	Observation group (*n* = 26)	Control group (*n* = 44)	t	*P*
The duration of postoperative mechanical ventilation (day)	6.23 ± 1.48	7.27 ± 1.72	2.570	0.012
The duration of postoperative antibiotic use (day)	8.28 ± 2.29	10.27 ± 2.13	3.673	0.001
Hospitalization (day)	12.37 ± 3.44	14.41 ± 3.28	2.469	0.016

## Discussion

With the improvement of prenatal diagnosis, an increasing number of fetuses with CDH can be diagnosed during pregnancy [12, 13]. Once the fetus was diagnosed with CDH in our hospital, after communicating with the parents and obtaining their consent, each child received tracheal intubation before umbilical dissection after birth; a gastric tube was placed through the nose, and mechanical ventilation respiratory support was given to strive for the best preoperative state. This not only avoids the aggravation of diaphragmatic hernia caused by gas entering the digestive tract because of crying, but also improves and maintains the oxygenation and respiratory conditions of the child. Due to the complex pathophysiological characteristics of CDH and malformations of the cardiovascular and digestive systems caused by the combined complications, anesthesia management of neonatal thoracoscopic-assisted and open diaphragmatic hernia repair is a significant challenge for the surgeon [14].

In most cases, the earlier the prenatal diagnosis of CDH is, the more severe the disease is. The LHR measured by B-mode ultrasound at different stages is an important indicator of the severity of diaphragmatic hernia [15, 16]. After ventilator treatment, children with low partial oxygen pressure and / or high partial carbon dioxide pressure in blood that is difficult to correct often need manual ventilation. The persistence or aggravation of pulmonary hypertension is also the cause of postoperative death. Use of positive pressure ventilator ventilation can reduce to occurrence of hypoxia and carbon dioxide accumulation in children, and pulmonary artery pressure is often gradually reduced. For children with persistent high pulmonary artery pressure that cannot be alleviated or hypoxia or carbon dioxide accumulation that cannot be improved, the possibility of intraoperative and postoperative hypoxia or carbon dioxide accumulation increases greatly, and the prognosis becomes much worse [17, 18].

Since the affected lung will be compressed by internal organs which occupy space during intrauterine development of CDH children, its development will be impaired to varying degrees; further, since the healthy lung is affected by mediastinal movement, its respiratory function will also be impaired to some extent [19, 20]. Previously publications have reported that pulmonary dysplasia on the affected side mainly manifests in different degrees of alveolar surface area reduction, the reduced number of bronchial trees, the decrease in pulmonary vascular inner diameter, and thickening of the median membrane, all of which result in pulmonary hypertension [21, 22]. Chest radiographs can also indicate to some extent the severity of lung involvement. After preoperative mechanical ventilation treatment and acid-base balance correction, some children can achieve better oxygenation and a more normal internal environment. In these cases pulmonary artery pressure does not increase rapidly, and thus they are more suitable for thoracoscopic surgery. Under the influence of artificial pneumothorax, the pressure-controlled breathing pattern often cannot fully meet the oxygenation needs of the children or maintain appropriate levels of carbon dioxide, so manual ventilation is required [23]. According to our experience, it is often necessary to increase the oxygen concentration, even to provide pure oxygen, and at the same time to increase the pressure of the escape valve in the respiratory circuit to maintain a inspiratory / expiratory ratio between 1:1.5 and 1:2 and a respiratory rate of about 25–35 times / min. Under these conditions, most children can maintain SpO_2_ > 90% and ETCO_2_ < 60 mmHg. In rare cases, higher air pressure may result in distension of the affected lung, affecting the surgical field. At this point, the operator must use the surgical hook to compress the lung, or reduce the pressure of the respiratory circuit to reduce the air in the lung. In this study, manual ventilation was used to correct hypoxia or carbon dioxide accumulation caused by various factors in intraoperative children, which achieved beneficial results. Maintaining the respiratory rate at 20–35 times / min, inspiratory peak pressure < 30 mmHg and the mean airway pressure < 6 mmHg improved the condition of hypoxia or carbon dioxide accumulation and allowed the operation to proceed smoothly.

The correction of acid-base balance and electrolyte disturbance before operation is also an important factor in ensuring the stability of vital signs during operation. According to literature reports, hypoxia, carbon dioxide accumulation and acidosis will further lead to pulmonary hypertension, weaken the role of hypoxic pulmonary vasoconstriction, and lead to hypoxia and carbon dioxide accumulation, forming a vicious cycle [24, 25]. Most of the children born with CDH underwent endotracheal intubation before umbilical cord dissection after birth and received an indwelling nasogastric tube for gastrointestinal decompression, which greatly reduced hypoxia or carbon dioxide accumulation after birth. Meanwhile, the correction of acid-base balance and electrolyte disorder were actively carried out, which provided optimized conditions for respiratory management and maintenance of vital signs in thoracoscopic surgery. Combined with the maintenance of respiratory system by means of intraoperative manual ventilation, good results were obtained in practical application.

This study is a retrospective study, and the severity of the disease in the two groups could not be selected and controlled, which is a limitation of this study. There was a statistical difference between the two groups in manipulative assistance in anesthesia, which may be caused by a difference in disease severity, and by the requirement for more manipulative ventilation assistance in open surgery. However, in general, there was no difference between thoracoscopy and laparotomy in anesthesia management, especially in respiratory management through manual ventilation, which provides some evidence and support for clinical operation and management strategies.

Another limitation of this study was a small sample size due to the limited time frame of this study. This may lead to errors in statistical analysis. Therefore, the sample size should be further expanded to minimize statistical errors, and to make the results of this study more convincing.

## Conclusion

In conclusion, intraoperative hemodynamic parameters of neonatal congenital diaphragmatic hernia repair under thoracoscope were more stable, and the time of mechanical ventilation, antibiotic use time and hospitalization time were shortened. The effect was better than that of open operation group.

## Data Availability

All data generated or analyzed during this study are included in this published article.

## Abbreviations

CDH: Congenital diaphragmatic hernia
ETCO_2_: End-tidal carbon dioxide
LHR: Lung to head ratio
SpO_2_: Value and pulse oxygen saturation
